# How to Control HTLV-1-Associated Diseases: Preventing *de Novo* Cellular Infection Using Antiviral Therapy

**DOI:** 10.3389/fmicb.2018.00278

**Published:** 2018-03-13

**Authors:** Amandine Pasquier, Sandrine Alais, Loic Roux, Maria-Isabel Thoulouze, Karine Alvarez, Chloé Journo, Hélène Dutartre, Renaud Mahieux

**Affiliations:** ^1^International Center for Research in Infectiology, Retroviral Oncogenesis Laboratory, INSERM U1111 – Université Claude Bernard Lyon 1, CNRS, UMR5308, Ecole Normale Supérieure de Lyon, Université Lyon, Lyon, France; ^2^Equipe Labellisée Ligue Nationale Contre le Cancer, Paris, France; ^3^Ecole Pratique des Hautes Etudes, Paris, France; ^4^CNRS UMR 7257, Architecture et Fonction des Macromolecules Biologiques, Aix-Marseille Université, Marseille, France; ^5^“Biofilm and Viral Transmission” Team, Structural Virology Unit, Department of Virology, CNRS UMR 3569, Institut Pasteur, Paris, France

**Keywords:** HTLV-1, antiviral therapy, proviral load, acyclic nucleoside phosphonates and thiophosphonates, AZT, prodrugs, cell–cell transmission

## Abstract

Five to ten million individuals are infected by Human T-cell Leukemia Virus type 1 (HTLV-1). HTLV-1 is transmitted through prolonged breast-feeding, by sexual contacts and by transmission of infected T lymphocytes through blood transfusion. One to ten percent of infected carriers will develop a severe HTLV-1-associated disease: Adult-T-cell leukemia/lymphoma (ATLL), or a neurological disorder named Tropical Spastic Paraparesis/HTLV-1 Associated Myelopathy (TSP/HAM). *In vivo*, HTLV-1 is mostly detected in CD4^+^ T-cells, and to a lesser extent in CD8^+^ T cells and dendritic cells. There is a strong correlation between HTLV-1 proviral load (PVL) and clinical status of infected individuals. Thus, reducing PVL could be part of a strategy to prevent or treat HTLV-1-associated diseases among carriers. Treatment of ATLL patients using conventional chemotherapy has very limited benefit. Some chronic and acute ATLL patients are, however, efficiently treated with a combination of interferon α and zidovudine (IFN-α/AZT), to which arsenic trioxide is added in some cases. On the other hand, no efficient treatment for TSP/HAM patients has been described yet. It is therefore crucial to develop therapies that could either prevent the occurrence of HTLV-1-associated diseases or at least block the evolution of the disease in the early stages. *In vivo*, reverse transcriptase (RT) activity is low in infected cells, which is correlated with a clonal mode of viral replication. This renders infected cells resistant to nucleoside RT inhibitors such as AZT. However, histone deacetylase inhibitors (HDACi) associated to AZT efficiently induces viral expression and prevent *de novo* cellular infection. In asymptomatic STLV-1 infected non-human primates, HDACi/AZT combination allows a strong decrease in the PVL. Unfortunately, rebound in the PVL occurs when the treatment is stopped, highlighting the need for better antiviral compounds. Here, we review previously used strategies targeting HTLV-1 replication. We also tested a series of HIV-1 RT inhibitors in an *in vitro* anti-HTLV-1 screen, and report that bis-POM-PMEA (adefovir dipivoxil) and bis-POC-PMPA (tenofovir disoproxil) are much more efficient compared to AZT to decrease HTLV-1 cell-to-cell transmission *in vitro*. Our results suggest that revisiting already established antiviral drugs is an interesting approach to discover new anti-HTLV-1 drugs.

## Introduction

Five to ten million individuals are infected worldwide by the oncogenic Human T-cell Leukemia Virus type 1 (HTLV-1) (Gessain and Cassar, 2012). This retrovirus is mainly present in Japan, Sub-Saharan Africa, the Caribbean region and Brazil. HTLV-1 is transmitted from mother-to-child through prolonged breast-feeding, by sexual contacts mostly from man to woman and by transmission of infected T lymphocytes through blood transfusion (Willems et al., 2017). HTLV-1 is mostly detected in CD4^+^ T-lymphocytes, and to a lesser extent CD8^+^ T-cells and dendritic cells *in vivo*. Interestingly, the differences in the number of HTLV-1-infected cells among distinct cell types are not due to the ability of HTLV-1 to infect these cells, but rather to its ability to persist and eventually transform the infected cells. Indeed, *in vitro*, human monocyte-derived dendritic cells are more susceptible to HTLV-1 infection than autologous CD4^+^ T-cells (Alais et al., 2015). In animal models, although it infects CD8^+^ T-cells and dendritic cells in the early phase of infection, HTLV-1 does not transform CD8^+^ T-cells (Rahman et al., 2010; Valeri et al., 2010; Kannian et al., 2013). Nevertheless, there is a strong correlation between HTLV-1 proviral load (PVL, i.e., the number of single integrated copies of the viral genome in cells), and the number and abundance of HTLV-1 infected CD4^+^ T-cells clones (Melamed et al., 2015), and the clinical status of the individuals (Gillet et al., 2013; Cook et al., 2014). A biomarker that would predict which carrier will develop an HTLV-1-associated pathology has not been described so far.

### Difficulties to Treat HTLV-1 Patients: Two Associated Diseases and Treatments

One to ten percent of infected carriers develop a severe HTLV-1-associated disease during their life: Adult-T-cell leukemia/lymphoma (ATLL), a CD4^+^ T lymphoproliferation of very poor prognosis with a mean survival time of 6 months in the acute form, or a progressive neurological disorder named Tropical Spastic Paraparesis/HTLV-1-Associated Myelopathy (TSP/HAM) (Yamano and Sato, 2012; Bangham et al., 2015). About 1000 cases of ATLL are diagnosed each year in Japan (Iwanaga et al., 2012). In the Martinique Island (French West Indies), a 14-year follow-up study allowed the detection of 123 TSP/HAM cases for only 400,000 inhabitants (Olindo et al., 2006). Because HTLV-1-infected cells are resistant to apoptosis-inducing agents, treatment of ATLL patients using conventional chemotherapy has very limited benefit (Utsunomiya et al., 2015). In Japan, when possible, ATLL patients undergo allogeneic hematopoietic stem cell transplantation (Utsunomiya et al., 2001). In Europe and United States, chronic and acute ATLL patients are treated with an anti-viral combination of recombinant interferon alpha (IFN-α) and AZT (zidovudine), combined in some cases with arsenic trioxide (El-Sabban et al., 2000; Nasr et al., 2011). It is worth noting that these results were convincingly confirmed by a world-wide meta analysis (Bazarbachi et al., 2010). However, this treatment is efficient only in a subset of patients, despite the efficacy of arsenic trioxide for inducing the death of HTLV-1-infected cells *in vitro* (El-Sabban et al., 2000; Mahieux and Hermine, 2005). While one report described the use of cyclosporin A for treating TSP/HAM patients with some benefits (Martin et al., 2012), treatment of these patients is in fact still an issue. Other drugs have been tried in clinic, although they generally have a very limited effect. As an example, a trial involving IFN-α had a modest but significant effect (Izumo et al., 1996). Different open trials [summarized by (Nakagawa et al., 1996)] have shown clinical benefit for glucocorticoids, followed by IFN-α, azathioprine and high-dose vitamin C. On the other hand, antiviral effects and/or a decrease in PVL, as well as immunomodulatory effects have been demonstrated for IFN-α (Saito et al., 2004; Rafatpanah et al., 2012), IFN-β (Oh et al., 2005), vitamin C (Moens et al., 2012), cyclosporine (Martin et al., 2012), danazol (Harrington et al., 1991), HDAC inhibitors (Lezin et al., 2007) in HAM/TSP *ex vivo* or *in vivo*.

### Viral Amplification Through Clonal Expansion in Addition to *de Novo* Infection: A Difference With HIV-1 Propagation

**FIGURE 1:**
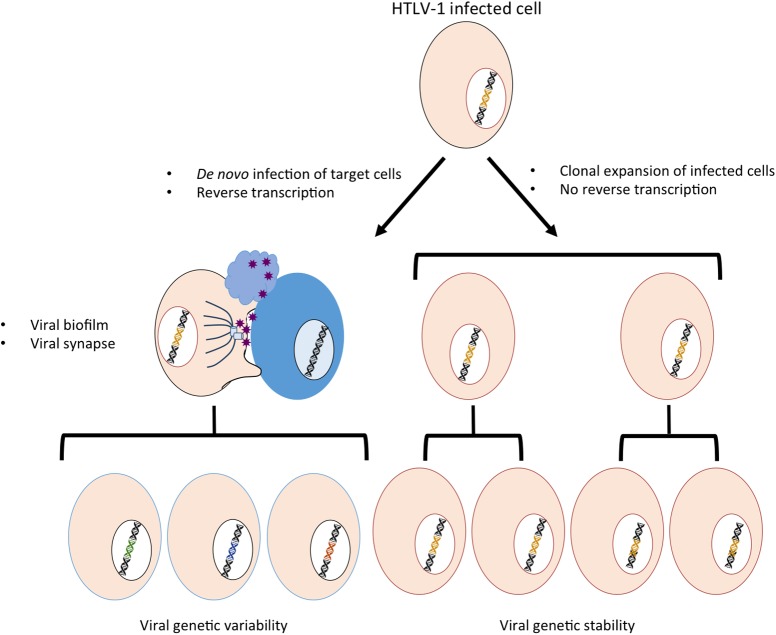
Schematic representation of the two modes of HTLV-1 amplification. Left: HTLV-1 transmission occurs through *de novo* cellular infection, which requires production of viral particles that are transmitted via viral biofilm and viral synapses and involves a reverse transcription step. The use of RT might lead to sequence variability. Right: HTLV-1 infection promotes clonal expansion of infected cells, associated with a stability in the proviral sequence.

Contrary to other viruses, HTLV-1 cannot be transmitted efficiently through cell-free viral particles. Using a cell-free experimental system, it was shown that compared to HIV-1, HTLV-1 had a low infectivity (at least 1000-fold lower luciferase activity) and that this was linked to some properties of the viral core and to post-entry processes that are still unclear (Derse et al., 2001). In contrast, HTLV-1 is efficiently transmitted following contacts between an infected donor cell and an uninfected target cell through the establishment of viral synapses and the transfer of viral biofilm (**Figure 1**, left part) (Igakura et al., 2003; Pais-Correia et al., 2010; Thoulouze and Alcover, 2011; Alais et al., 2015). Of note, cell-associated viral transmission of HIV-1 through nanotubes, filopodes or viral synapses is also more efficient than the cell-free infection protocol that is commonly used in *in vitro* experiments (Jolly and Sattentau, 2005; Sherer et al., 2007; Sowinski et al., 2008; Rudnicka et al., 2009; Zhong et al., 2013).

HTLV-1 infection then leads to the clonal expansion of infected cells (**Figure 1**, right part) (Wattel et al., 1995; Bangham et al., 2014; Turpin et al., 2017; Watanabe, 2017). Because the reverse transcriptase (RT) is not involved in replication by clonal expansion, this phenomenon may explain the very low genetic variability of the virus despite the low fidelity of its RT (Mansky, 2000). Interestingly, HIV-1 clonal expansion also occurs (Maldarelli et al., 2014; Boritz et al., 2016) and has been suggested to allow the virus to overcome antibody neutralization and surface retention by the tetherin restriction factor (Zhong et al., 2013), although the consequences of such a phenomenon remain to be fully understood.

### Targeting Viral Replication With the Use of Antivirals: The HIV Example

Nowadays, 25 antiretroviral agents classified in six classes have been approved to treat HIV infections (Cihlar and Fordyce, 2016). The antiretroviral therapy (cART) involves combinations of drugs to achieve maximal response and is generally composed of two nucleoside/nucleotide reverse transcriptase inhibitors (NRTIs) and a third active antiviral from a different class (NNRTI: non-nucleosidic RT inhibitor, INSTI: integrase inhibitor, PI: protease inhibitor, EI: entry inhibitor).

Nucleotide reverse transcriptase inhibitors were the first class of compounds to be used in HIV therapy, by the approval of zidovudine (AZT) in 1987 (Yarchoan et al., 1986), initially discovered as potent anti-cancer agent (Furmanski et al., 1980). NRTIs are compounds that become active after being phosphorylated into their triphosphate forms, in a process that involves three distinct phosphorylation steps catalyzed by cellular kinases. While they are generally poor substrates for cellular polymerases, triphosphorylated NRTIs compete with natural triphosphate nucleotides for incorporation into growing viral DNA by HIV RT (Furman et al., 1986), resulting in DNA chain termination by blocking further DNA extension (Mitsuya and Broder, 1987).

Historically, AZT and ddI (didanosine) were first shown to block HIV replication in T cells (Mitsuya et al., 1985) and subsequently were shown to suppress HIV replication in monocytes and macrophages *in vitro* (Perno et al., 1988). Other nucleoside analogs without oxacyclopentane sugar moiety, as well as 2′,3′-dideoxynucleosides such as d4T (Balzarini et al., 1987), or carbocyclic nucleosides such as Abacavir (ABC) (Daluge et al., 1997), were then identified to be active against HIV, resulting in the emergence of structure activity relationship (SAR) knowledge (Balzarini et al., 1987). However, nucleoside analogs were not equivalent in either activity or toxicity profiles when used as therapeutic drugs *in vitro* and *in vivo* (Mitsuya et al., 1990), and in particular in their capacity to be activated through phosphorylation in cells. Indeed, during this indispensable process, the first phosphorylation step is often rate-limiting and thus an important bottleneck that limits NRTI efficacy in cells.

To circumvent this drawback, acyclic nucleoside phosphonates (ANPs) have revolutionized the antiviral drug field (Holy, 2003; De Clercq and Holy, 2005). ANPs are nucleotide analogs with a phosphonate group attached to the nucleoside moiety, which require only two phosphorylation steps to be converted into their fully phosphorylated active form (Sharma et al., 2004). The anti-HIV properties of Tenofovir (PMPA, 9-[(R)-2-(Phosphonomethoxy)Propyl]Adenine) were first described in Balzarini et al. (1993) and 8 years later, the oral prodrug, namely Tenofovir disoproxil (TDF) was licensed for clinical use for the treatment of HIV infection. As such, TDF has remained a cornerstone for 14 years and has been replaced recently by a new oral prodrug of the same compound, namely Tenofovir Alafenamide (TAF), which allows a better pharmacokinetic profile in patient fluids (Ray et al., 2016).

Undesirable emergence of viral resistance, toxicity, drug-related adverse effects and drug-drug interactions associated with treatment using NRTIs have challenged the antiviral therapy. Over the last 30 years, it has led to the discovery and approval of several new compounds (Cihlar and Fordyce, 2016) with new ones still in ongoing development (Sax et al., 2015). This intense antiviral research provided the clinic with different anti-HIV active drugs that are now used in combination and have markedly decreased mortality and morbidity from HIV-1 infections in the developed world (Palella et al., 1998). This illustrates that the effort to provide the clinic with new drug regiments results in successful therapies that benefit to patients.

In 2017, the NRTIs approved by the FDA for the treatment of HIV-1 include Retrovir^®^ (Zidovudine, AZT, 1987), Videx^®^ (didanosine, ddI, 1991), Zerit^®^ (stavudine, d4T, 1994), Epivir^®^ (lamivudine, 3TC, 1995), Combivir^®^ (AZT + 3TC, 1997), Ziagen^®^ (abacavir sulfate, ABC, 1998), Trizivir^®^ (AZT + 3TC + ABC, 2000), Viread^®^ (tenofovir disoproxil fumarate, TDF, 2001), Emtriva^®^ (emtricitabine, FTC, 2003), Epzicom^®^ (3TC + ABC, 2004), Truvada^®^ (TDF + FTC, 2004), Atripla^®^ (TDF + FTC + Efavirenz (NNRTI), 2006), Complera^®^ [TDF + FTC + Elvitegravir (INSTI) + booster, 2011], Triumeq^®^ [3TC + ABC + dolutegravir (INSTI), 2014], Genvoya^®^ [FTC + Tenofovir Alafenamide (TAF) + Elvitegravir (INSTI) + booster, 2015], Odefsey^®^ [FTC + TAF + rilpivirine (NNRTI), 2016], Descovy^®^ (FTC + TAF, 2016). Hivid^®^ (zalcitabine, ddC, 1992) was discontinued due to genotoxicity. This antiviral success prompted clinician and researchers to test whether some of the anti-HIV drugs, and especially the acyclic phosphonates ones, could also be active against infections in which viral replication use a viral DNA polymerase enzyme, such as HBV, HSV, EBV, or CMV (De Clercq and Holy, 2005). Interestingly, although the acyclic phosphonate-including class has a broad-spectrum efficacy against several divergent DNA virus families, each acyclic phosphonate within the class has its own specificity. For example, HPMPC is able to inhibit the replication of *Papovaviridae*, *Adenoviridae* and some viruses from the *Herpesviridae* family, while PMEA and PMPA are able to inhibit *Hepadnaviridae* and *Retroviridae*, and PMEA and HPMPC are able to inhibit the replication of several, but not all *Herpesviridae* (De Clercq and Holy, 2005). Thus, some inhibitors first described as active against HIV may have a broader antiviral spectrum, even on viruses belonging to different classes, strongly supporting the concept of re-positioning of existing drugs.

### HIV-NRTI Repositioning on HTLV-1

Because replication of both HIV-1 and HTLV-1 retroviruses (whether it is a frequent step or not, as discussed above) requires RNA reverse transcription performed by phylogenetically related RTs, it is tempting to hypothesize that some anti-HIV-1 drugs targeting HIV-1 RT might also be efficient against HTLV-1 RT. Indeed, using cellular assays of viral transmission, it was shown that AZT, 3TC, phosphonated carbocyclic 2′-Oxa-3′-Aza-nucleoside and Tenofovir each reduced HTLV-1 transmission through cell-cell contact (see **Table 1**) (Macchi et al., 1997; Zhang et al., 2001; Balestrieri et al., 2002, 2005, 2008; Chiacchio et al., 2005, 2007; Pais-Correia et al., 2010). Furthermore, *in vitro* RT assays using HTLV-1 virions confirmed that these NRTIs target the enzymatic RT activity of HTLV-1 (Garcia-Lerma et al., 2001; Balestrieri et al., 2005, 2008; Macchi et al., 2011). The first inhibitory values were obtained using a test that is not quantitative but allows only to determine the drug efficiency (Yamamoto et al., 1996). However, a clinical trial involving AZT as a putative HTLV-1 RT inhibitor, showed only a very limited efficacy in reducing HTLV-1 PVL and no immunological or clinical responses could be demonstrated (Taylor et al., 2006). Furthermore, other data suggested that AZT could in fact inhibit telomerase and lead to cell death, irrespective of the infected status of treated cells (Datta et al., 2006).

**Table 1:** Summary of reported inhibition tests using NRTI in cell-to-cell transmission **(A)** or in *in vitro* RT assays **(B)**.

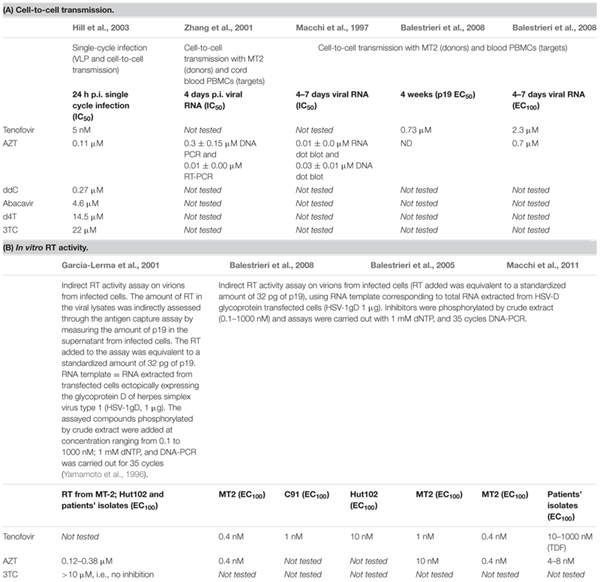

*IC_50_ is inhibitory concentration 50%, EC_50_ is effective concentration 50%. EC_100_ is effective concentration 100%*

A number of studies were designed to decipher why nucleoside analogs, contrary to HIV-1, are so inefficient against HTLV-1. One obvious reason, already mentioned, is the pseudo-latency of the virus (Taylor and Matsuoka, 2005; Taylor et al., 2006). Indeed, in chronically infected asymptomatic carriers, HTLV-1 does not frequently replicate by reverse transcription but rather replicates through clonal expansion of the infected cells (Wattel et al., 1995; Gillet et al., 2011, 2017). Another explanation lies in the mode of viral cell-to-cell transmission. As discussed above, HTLV-1 transmission through cell-free viral particles does not seem efficient. In contrast, cell-to-cell transmission mostly occurs through cell–cell contacts between an infected cell and an uninfected target (Derse et al., 2001; Ceccaldi et al., 2006; Mazurov et al., 2010; Alais et al., 2015). HTLV-1 transmission after cell-to-cell contacts is thought to lead to a high multiplicity of infection and to drug resistance, as was previously described in the case of HIV (Sigal et al., 2011; Zhong et al., 2013). Interestingly, while AZT is indisputably recognized as an efficient HIV-1 replication inhibitor, reported IC_50_ are consistently higher when inhibition assays are performed using cell-to-cell infection settings instead of cell-free virus (Sigal et al., 2011; Agosto et al., 2014), suggesting that cell-to-cell transmitted viruses have an inherent resistance to reverse transcription inhibitors, which may not be linked to the molecular enzymatic resistance of the RT. Thus, efficacy of AZT or other NRTIs against HTLV-1 might be further limited by the almost exclusive cell-to-cell transmission mode of the virus.

Several parameters might further account for the discrepancies between results obtained *in vitro* and *in vivo*. First, the efficacy of AZT against HTLV-1 as measured by the IC_50_ might be dependent on the experimental settings. This is illustrated by a study that reported a 1000-fold lower efficacy of compounds when used in cell-to-cell transmission assays (inhibition range of 1 μM) compared to *in vitro* RT inhibition assays performed on virions from supernatant of infected cells (inhibition range of around 1 nM) [**Table 1**, (Balestrieri et al., 2008)]. Thus, IC_50_ determined *in vitro* or in cellular assays may not be relevant to what occurs *in vivo*. Furthermore, sequence variation within the RT gene, in addition to its influence on replication efficiency (Mitchell et al., 2007), might also be responsible for different sensitivity of RT from virions produced by different HTLV-1 infected cells lines (Balestrieri et al., 2008) or RT derived from patient isolates (Macchi et al., 2011) to NRTIs (**Table 1**). Even if inhibition is measured in cell–cell transmission assays using the same infected donor cells, the use of different target cells might explain the variability in NRTI efficacy. For example, AZT efficacy was measured on HTLV-1 transmission after co-culture of chronically infected MT2 cells with primary cord PBMCs (Zhang et al., 2001) or adult blood PBMCs (Macchi et al., 1997; Balestrieri et al., 2008) (**Table 1**). In these assays, AZT was estimated to inhibit HTLV-1 transmission after 4–7 days of culture, as measured by viral DNA or viral RNA detected in primary PBMCs. AZT IC_50_ values were 0.03 ± 0.01 and 0.01 ± 0.00 μM for DNA and RNA, respectively, in PBMCs and 0.30 ± 0.15 and 0.01 ± 0.00 μM for DNA and RNA in cord blood, respectively (**Table 1**). The lower susceptibility to AZT inhibition of PBMCs from adult blood highlights that besides the direct effect of AZT on RT activity in virions released from MT2 infected cells (**Table 1**), other mechanisms exist in the target cells that modulate the efficacy of inhibition. This could include drug uptake and intracellular phosphorylation that have been shown to be cell-type dependent (Deville-Bonne et al., 2010; Roux et al., 2013), or RT activity in the target cell that could differ depending on the cellular environment. Very little is known regarding RT activity in cells during the early steps of the viral cycle. Yet, because cell-to-cell infection is much more efficient than cell-free infection, it is possible that either a higher number of virions is delivered to the target cell during cell-to-cell contact or that contact-dependent signals are required to enhance virus replication. Such signals could also modulate the sensitivity of target cells to RT inhibition, in a target cell-specific manner.

Of note, although co-culture assays probably mimic HTLV-1 transmission *in vivo*, inhibition is measured after several days of culture. This long-term culture of infected cells could select for T-cells susceptible to infection and able to survive after HTLV-1 infection, due to the immortalization potency of the virus. Furthermore, several rounds of infection could occur that may not be suitable for quantitative analysis of infection and replication. Alternative experimental systems have been developed to measure HTLV-1 spread after a single-cycle replication (Derse et al., 2001) and to measure viral inhibition using NRTI (Hill et al., 2003) (**Table 1**). This elegant system [reviewed in (Aloia et al., 2013)] is based on virus-like particles (VLPs) that contain HTLV-1 RT and that deliver a surrogate retroviral genome designed to express only a reporter gene product in infected cells. These VLPs are generated by co-transfecting cells (usually adherent cell lines such as HEK293T) with a packaging plasmid (allowing the expression of HTLV-1 structural, enzymatic and regulatory proteins under the control of a CMV promoter), a transfer reporter vector and an envelope-encoding plasmid [reviewed in (Derse et al., 2001)]. In these settings, the inhibition properties of several nucleoside analogs were determined (**Table 1**) and results confirmed AZT IC_50_ to be around 0.1 μM (Hill et al., 2003). It is worth noting that efficiencies of all compounds tested against HTLV-1 were at least 100-fold lower than that tested against HIV-1, further suggesting that although both RTs have similar sequence and enzymatic organization, the catalytic properties of the two RTs are different and/or differently regulated by the cellular environment of the infected cells. Moreover, although useful to monitor single-cycle infections, this system uses VLPs that may be different from WT virions and more importantly, it uses transfected HEK293T donor cells that are very different from the natural infected T-cells expected to transfer the virus *in vivo*. Knowing that cell–cell contacts may regulate HTLV-1 RT activity, the nature of the infected cell used to transfer the virus is expected to modulate the infectivity of the transferred virions as well as the efficacy of NRTIs.

### Strategies to Control HTLV-1 PVL *in Vivo*: Replication Reactivation and Use of NRTIs

As discussed above, HTLV-1 pseudo-latency renders this retrovirus usually insensitive to RT inhibitors. Interestingly, viral expression and RT-dependent *de novo* cellular infection can be induced using histone deacetylase inhibitors (HDACi) such as valproic acid (VPA) (Lezin et al., 2007; Olindo et al., 2011). Importantly, long-term treatment of HTLV-1-infected patients with VPA is proven to be safe (Lezin et al., 2007; Olindo et al., 2011). In an earlier work conducted in non-human primates naturally infected with Simian T-Lymphotropic Virus type 1 (STLV-1), the simian equivalent of HTLV-1, we tested a combination of VPA, used to increase viral replication, and AZT, used to inhibit *de novo* cellular infection by targeting the RT activity (Afonso et al., 2010). Because the PVL is higher in TSP/HAM patients than in asymptomatic carriers and can therefore be considered as a prognostic marker (Yakova et al., 2005), we selected asymptomatic STLV-1-infected animals with a high PVL (Afonso et al., 2010). Our results showed for the first time that the AZT/VPA combination leads to a very strong and significant PVL decrease in all tested animals. However, STLV-1 PVL rebounded after the treatment was stopped, thus demonstrating that the virus had not been cleared during the treatment, a disappointing result reminiscent of what is described for HIV-1. As for HIV-1, this rebound of HTLV-1 viral load suggests (i) the existence of a putative viral reservoir inaccessible to the drugs, or (ii) that AZT is not efficient enough to block *de novo* HTLV-1 infection that occurs at a level below the detection threshold, or (iii) that viral escape mutants are selected during treatment, leading to resistance to AZT, although this is unlikely given that resistance mutation were not reported in human patients undergoing AZT. Nevertheless, these results demonstrate that preventing *de novo* infection is an option for controlling the virus and foster the use of more efficient drugs that would better control HTLV-1 dissemination within infected individuals.

Here we show a series of results obtained with already tested drugs as well as with newly compounds. We report for the first time that bis-POM-PMEA (adefovir dipivoxil) and bis-POC-PMPA (tenofovir disoproxil) are much more efficient compared to AZT to decrease HTLV-1 cell-to-cell transmission *in vitro*.

## Materials and Methods

### Cells

Jurkat, Jurkat-LTR-Luc (Pais-Correia et al., 2010), and HTLV-1-infected T-cell line (C91PL) were maintained in RPMI 1640 medium supplemented with 10% fetal calf serum and penicillin-streptomycin (100 μg/ml, Gibco Life Technology). All cells were grown at 37°C in 5% CO_2_. Jurkat-LTR-Luc cells, stably transfected with a plasmid encoding luciferase, under the control of the HTLV-1 long terminal repeat (LTR) promoter are maintained under hygromycin (450 μg/ml, Sigma) selection.

### Drugs

AZT, d4T, 3TC, tenofovir alafenamide, tenofovir disoproxyl and tenofovir disoproxyl fumarate were purchased from Selleckchem. PMPA, PMEA were provided by Dr. Alvarez (AFMB, Marseille). S-PMPA, S-PMEA, bis-POC-S-PMPA, bis-POM-PMEA, and bis-POM-S-PMEA were synthesized and provided by Dr Alvarez (AFMB, Marseille). All drugs were resuspended in DMSO or water according to the furnisher, aliquoted and stored at -80°C until used. Aliquots were used only once to avoid drug degradation under thawing-freezing cycles.

### Inhibition of Viral Transmission Test

Jurkat reporter cells (JK-LTR-Luc, 50.000 cells) were seeded in 96-well plates and treated for 18 h in presence or absence of the different drugs (1–50 μM, diluted in medium). Then, cells were washed once and HTLV-1-infected cells (C91PL, 10.000 cells) or control cells (Jurkat, 10.000 cells) were added together with or without an additional ½ dose of drugs, and the co-culture was resumed for 24 h. Cell viability was measured by trypan blue exclusion counting. The mortality index was determined as the percentage of dead cells in each condition normalized to the percentage of dead cells in the control condition set to 1. A mortality index higher than 1.5 signs drug toxicity. Reporter activities were assayed using the luciferase reporter assay system (Promega). Luciferase activity was normalized according to the amount of total proteins in the co-culture determined by Bradford (Bio-Rad). Results were then expressed as a percentage of control in the absence of drugs.

### Statistical Analysis

One-way analysis of variance (ANOVA) with Bonferroni’s *post hoc* multiple comparison test was used to determine statistically significant differences. Differences were considered significant if the *p*-value was < 0.05.

## Results

### New Strategies for the Repositioning of Anti-HIV Inhibitors as Efficient Anti-HTLV-1 Drugs

High-throughput screening efforts against HIV-1 followed by structure-based drug design have allowed the discovery of several drugs that are more potent than AZT and related nucleoside analogs, and are still ongoing (Taylor et al., 2016). Highly active non-nucleoside inhibitors eliciting hardly any resistant mutants have been designed when purified target enzymes (RT, protease, integrase) were made available, providing essential structural and functional models. In strong contrast, discovery of anti-HTLV-1 specific drugs is currently significantly impeded by the unavailability of purified viral enzymes. In addition, and as stated above, because the virus is almost exclusively transmitted through cell–cell contacts, cell infection cannot be easily induced *in vitro* using cell-free particles as a source of infectious viruses (Alais et al., 2015). This further challenges the development of drug screening protocols. Since HIV-1 and HTLV-1 use very similar mechanisms of reverse transcription, we hypothesized that anti-HIV-1 NRTIs might be efficient against HTLV-1 RT. NNRTIs, which are highly specific to HIV-1 RT, were thus excluded from our screen.

**FIGURE 2:**
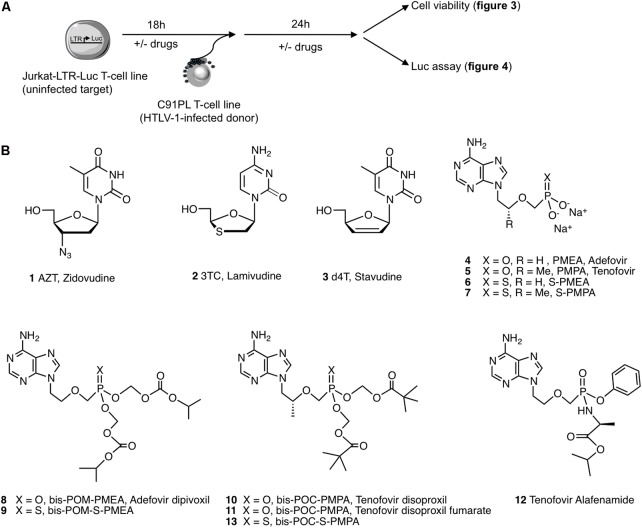
Experimental assay and chemical structures of compounds used in this study. **(A)** Schematic representation of the experimental procedure. Jurkat-LTR-Luc [reporter cells for HTLV-1 productive infection in which expression of the luciferase gene is under the control of the viral Tax-dependent promoter (LTR)] are pretreated or not with drugs for 18 h before their co-culture with C91PL (HTLV-1 infected T-cell line) for 24 h. Cell viability and luciferase activity are then measured. **(B)** AZT (1) is used as the reference drug during all experiments, while 3TC (2) and d4T (3) are used as controls; the acyclic nucleoside phosphonate PMEA (4), its thio-derivative S-PMEA (6) and their prodrugs (8) and (9), respectively; the acyclic nucleoside phosphonate PMPA (5), its thio derivative S-PMPA (7) and their pro-drugs (10), (11), (12), and (13), respectively. Structures were drawn using ChemDraw. The molecule names (when available) are indicated under the structure.

We thus set up an *in vitro* cell co-culture assay that allows for the monitoring of HTLV-1 *de novo* infection in LTR-luciferase reporter Jurkat T-cells using HTLV-1 chronically infected C91PL T-cells as a source of virus (Alais et al., 2017) (**Figure 2A**). This system was used to test the efficacy of several NRTIs known to inhibit HIV-1 RT (**Figure 2B**). In addition, we selected pro-drugs of these NRTIs and evaluated their efficacy and toxicity. AZT (1) was used as a positive control. 2′,3′-dideoxynucleosides such as 3TC (2) and d4T (3) were also used as controls (**Figure 2B**). Because highly variable data are reported in the literature on the effectiveness of these compounds (**Table 1**), we also included them to calibrate the stringency of our assay. While the ANP Tenofovir (PMPA, 5) has been tested against HTLV-1 previously, its congener Adefovir (PMEA, 4) has never been evaluated on HTLV-1 so far, although its efficiency to inhibit HIV and HBV infections is described (De Clercq and Holy, 2005). Both compounds were included in our screen. We also included new ANP analogs, named thiophosphonates, S-PMEA (6) and S-PMPA (7), which have shown potent activity against HIV and HBV infections (Barral et al., 2011; Roux et al., 2013; Priet et al., 2015).

As discussed above, ANPs are NRTIs that contain a stable phosphonate group. After penetration into the target cells, ANPs are phosphorylated into their diphosphate forms which serve as substrates for RT and thus exert the antiviral effect. Because ANPs are negatively charged at physiological pH, they are less efficient in passing through cell membranes than neutral, lipophilic species. To address this issue, these compounds can be prepared as prodrugs. The activity of ANPs is dramatically increased by the use of their biolabile prodrugs: Adefovir dipivoxil (Hepsera^®^) was licensed for the treatment of chronic HBV infections during several years (Merle and Trepo, 2001) and Tenofovir is commercialized as two prodrugs, which are much more potent than their parent counterparts.

We therefore also evaluated Adefovir dipivoxil (bis-POM-PMEA, 8), its thiophosphonate equivalent bis-POM-S-PMEA (9), Tenofovir disoproxil (bis-POC-PMPA, 10), Tenofovir disoproxil fumarate (bis-POC-PMPA, 11), Tenofovir alafenamide (12) and the thiophosphonate congener bis-POC-S-PMPA (13).

**FIGURE 3:**
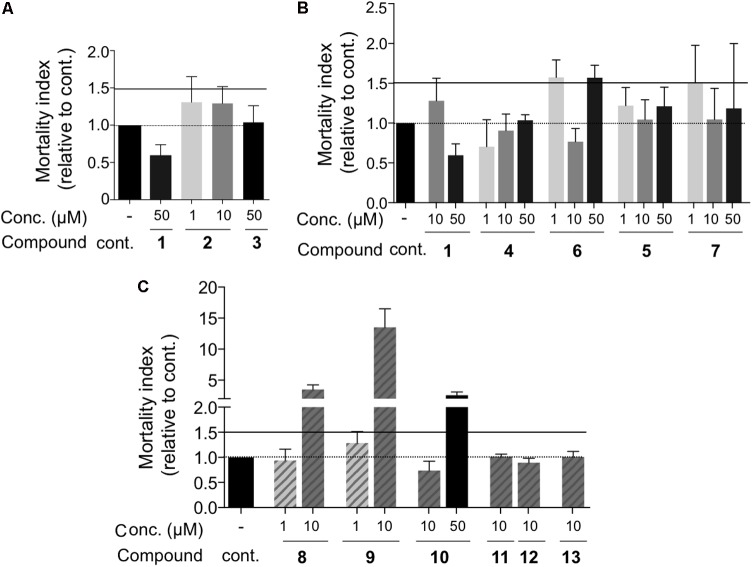
Control of cell viability during drug treatment. Jurkat-LTR-Luc reporter cells were treated for 42 h and the proportion of dead cells was determined by trypan blue dye exclusion test. Data were normalized to the proportion of dead cells in untreated control conditions, set to 1, to obtain a mortality index. A mortality index higher than 1 indicate a drug-induced mortality. Drugs were used in the following assays if the mortality index was below 1.5. Drug concentration and compound number are indicated. Results were grouped based on the drug family: **(A)** nucleoside analogs, **(B)** acyclic nucleoside phosphonates, **(C)** prodrugs of acyclic nucleoside phosphonates. No significant differences were observed (one-way ANOVA), *n* = 2–4 independent experiments with triplicate samples.

**FIGURE 4:**
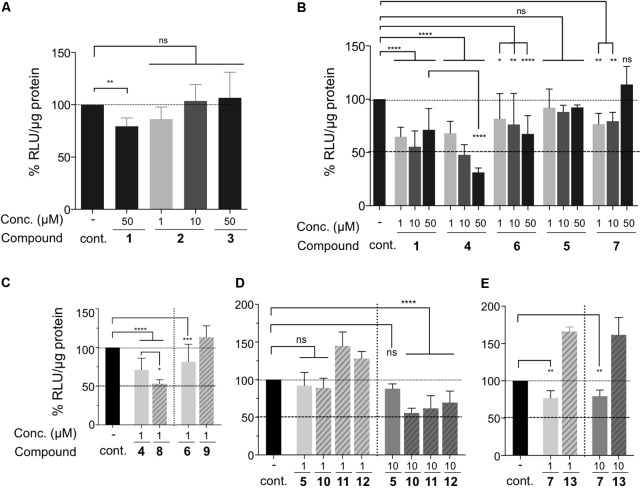
Inhibition efficacy of drugs on HTLV-1 cell-to-cell transmission. **(A–E)** Luciferase activity was normalized to protein concentration (in μg, quantified by Bradford assay) and expressed as percent of untreated (control) cells set as 100%. Drug concentrations and drug names are indicated. Results were grouped based on the drug family: **(A)** AZT (1), 3TC (2) and d4T (3) nucleoside analogs, **(B)** AZT (1) and acyclic nucleoside phosphonates, **(C)** PMEA and S-PMEA and their prodrugs, **(D)** PMPA and a series of PMPA pro-drugs, **(E)** S-PMPA and its pro-drug. Statistical differences were determined using one-way ANOVA (ns, not significant, ^∗^*p* < 0.05, ^∗∗^*p* < 0.01, ^∗∗∗^*p* < 0.001, ^∗∗∗∗^*p* < 0.0001, *n* = 2–4 independent experiments with triplicate samples).

First, we tested whether these compounds had any impact on target cell viability (**Figure 3**). The concentration of these drugs varied between 1 and 50 μM. The viability of treated cells was represented relative to that of the untreated cells, set to 1. An index > 1 means that the mortality of the treated cells is higher than that of the untreated cells. We considered that cell viability was impaired if the index was >1.5 and thus excluded all the drug concentrations leading to a mortality index > 1,5. None of the drugs, i.e., AZT (1), 3TC (2), d4T (3) (**Figure 3A**) as well as the PMEA (4–6) and PMPA (5–7) families had any significant effect on cell viability, i.e., their mortality index was lower than 1.5 (**Figure 3B**). As already reported (Roux et al., 2013), Bis-POM-PMEA (8) and Bis-POM-S-PMEA (9) were toxic when used at 10 μM (**Figure 3C**), as well as BIS-POC-PMPA (10) when used at 50 μM (**Figure 3C**). Thus, these concentrations were excluded for the subsequent evaluation of their ability to prevent HTLV-1 transmission to reporter T-cells after co-culture with infected donor cells (**Figure 4**). LTR-luciferase Jurkat reporter T-cells were used as target cells (Alais et al., 2017). Given that Tax is absent from the viral particle, transcription from the viral Tax-dependent LTR requires productive infection and Tax expression in the target cells, which can occur after contact with the infected donor cells through the establishment of viral synapses or transfer of viral biofilm (Pais-Correia et al., 2010). Interestingly, this experimental system does not rely on cell-free infection and is therefore closer to *in vivo* viral transmission.

Consistent with previous reports, AZT (1) had a significant but limited effect on HTLV-1 transmission, even at the high concentration of 50 μM (inhibition of 10–20% **Figures 4A,B**), while 3TC (2) had no effect, independently of the concentration used (**Figure 4A**). Surprisingly, AZT (1), had the same significant inhibitory activity for all the doses tested, without any dose-dependent effect (**Figure 4B**). d4T (3) also had no effect (**Figure 4A**), suggesting that previously published cell-free experiments using virion-associated enzymatic RT assays might not be relevant for determining that a drug has an anti-HTLV-1 effect (Garcia-Lerma et al., 2001). We then tested phosphonate PMEA (4) and PMPA (5) (**Figure 4B**) and compared their effect to that of AZT. Contrary to AZT, PMEA (4) had a statistically significant dose-dependent inhibitory activity (**Figure 4B**). When used at 50 μM, PMEA was statistically more efficient than AZT (70% vs. 30% of viral transmission decrease, respectively, *p* < 0.0001). Of note, PMEA’s active concentration on HTLV-1 was much higher than the dose reported to inhibit HIV-1 cell-free transmission, which is around 5 μM (Barral et al., 2011). Importantly, it is worth noting that when HIV-1 inhibition assays were performed using cell-to-cell infection instead of infection with cell-free virus, reported IC_50_ were also consistently higher (Sigal et al., 2011; Agosto et al., 2014). PMPA (5) did not show any inhibitory effect compared to control, AZT (1) or PMEA (4), independently of the concentration used (**Figure 4B**). This is somehow different from a previous report which showed that overnight Tenofovir treatment (1 μM) before co-culture had some effect on viral transmission from chronically-infected MT2 cells to PBMCs, while the same product at 0.01 μM had no effect (Balestrieri et al., 2005). We also tested S-PMEA (6) and S-PMPA (7) (**Figure 4B**), which showed a limited effect for (6) at the highest concentrations, yet not significantly better than (1) and significantly worse than (4). Thus, modifying PMEA into the thio-derivative S-PMEA (6) results in a strong decrease in HTLV-1 transmission inhibition [compare (4) vs. (6)], consistent with the results on HIV replication for which IC_50_ is threefold higher than that of the parental derivative (Barral et al., 2011). Interestingly, modifying PMPA into its thio-derivative (7) resulted in a limited but significant better inhibition than 5, at 1 and 10 μM (25% of inhibition *p* < 0.01), an effect that is lost when the concentration is increased to 50 μM (**Figure 4B**), probably due to the higher toxicity of 7 at 50 μM (**Figure 3B**).

We then tested ANP pro-drugs since they were reported to display an active concentration that was better than that of their parent compound due to the higher ability in passing through cell membranes (**Figure 4C**). bis-POM-PMEA (8) showed a statistically better, although modest, inhibition of LTR activation at 1 μM than the parent compound PMEA (4) tested at the same concentration (compare **Figure 4C**). Although S-PMEA (6) had a significant yet modest inhibitory effect at 1 μM (20% of inhibition *p* < 0.001, **Figure 4C**), its bis-POM modification (9) results in a loss of this effect when used at the same concentration. This suggests that although the cell-membrane penetration is increased, it does not translate into a better efficacy of the compound. This could be explained by the higher toxicity of the pro-drug (6) at 1 μM (**Figure 3C**), which within the 42 h of treatment could impair the metabolism of the pro-drug after its penetration into the cells. Higher concentration could not be used because of compound 9 toxicity (see **Figure 3C**).

For the PMPA compound family, no effect was observed when pro-drugs (10, 11, or 12) were used at 1 μM. This is consistent with the absence of inhibition observed with the parent PMPA (5) compound (**Figure 4D**). However, when used at 10 μM, prodrugs (10–12) had a higher effect than PMPA (5) itself and induced a significant reduction (50%, *p* < 0.0001) of HTLV-1 replication.

Finally, bis-POC-S-PMPA (13) did not show any improvement over S-PMPA (7) and even had a statistically significant positive effect on viral transmission (**Figure 4E**). This could be explained by the lower de-protection rate of (13) in cell, thus leading to a lower concentration of active S-PMPA within cells (Roux et al., 2013). Surprisingly, the pro-drug effect was lower than expected from the results observed in HIV tests in which IC_50_ are decreased by at least 120-fold (Roux et al., 2013) by bis-POC or bis-POM modifications. This suggests that in our settings, the rate of pro-drugs metabolism and their subsequent use as substrates by HTLV-1 RT might be very low, although they could increase over time, leading to a potential better inhibition.

Altogether, our results show that PMPA (5) is not active when HTLV-1 infection occurs through cell–cell contacts, although (5) inhibits HTLV-1 RT in *in vitro* enzymatic assays (see **Table 1**). However, we demonstrate that several pro-drugs of PMPA are efficient (10, 11, 12), confirming earlier studies (Macchi et al., 2011) and suggesting that they could be used in clinical trials. More importantly, our results extend the inhibitory potential of phosphonate derivatives by showing that PMEA (4) is more efficient than PMPA (5) to block HTLV-1 viral transmission, without any associated-toxicity. In addition, PMEA pro-drug (8) is also efficient, and at a 10-fold lower concentration than all PMPA pro-drugs. However, its associated-toxicity might compromise its use for clinical trial. Finally, the thio-modification of PMEA (6) does not increase PMEA (4) inhibitory potential, while PMPA thio-modification (7) increases PMPA (5) efficiency, although this effect is lost for the pro-drug (13).

## Discussion

HTLV-1-induced diseases, i.e., ATLL and TSP/HAM, are of bad prognosis. Despite the early discovery of HTLV-1 more than 30 years ago, and despite the outstanding genetic stability of the virus (which could be a topic of interest for the vaccine industry), specific anti-HTLV-1 drugs have not been developed yet. In addition, and contrary to the HIV-1 field, asymptomatic HTLV-1-infected individuals are not treated, although a high PVL is established to be a valuable risk factor that could be used as a biomarker to identify individuals at risk for developing HTLV-1-associated diseases. Rather, patients are treated once they develop the disease, which is probably too late. Clonal populations of infected T-cells are detected *in vivo*, suggesting that the virus mostly replicates through clonal expansion (Bangham et al., 2014), although viral expression, potentially leading to viral production, may occur upon metabolic changes occurring in protected body areas (Kulkarni et al., 2017). In addition, HTLV-1 does not replicate only through clonal expansion given that AZT as an *in vivo* effect in asymptomatic individuals (Afonso et al., 2010).

Anti-HIV-1 drugs were first tested using cell-free infection systems in which it is easy to quantify viral production. This is the reason why reverse-transcriptase inhibitors have also been tested using HTLV-1 purified virions (Garcia-Lerma et al., 2001; Balestrieri et al., 2005, 2008), or after co-culture between MT2 infected cell line and PBMCs from normal blood donor (Macchi et al., 2011). This is, however, an issue for translating results into the clinic. Indeed, when it occurs, HTLV-1 transmission goes through cell–cell contacts rather than through cell-free viral particles, a transmission mode that was proven to increase viral resistance to drugs in the HIV-1 situation. Previously described co-culture assays for drug screening used MT2 cells as donor cells (Macchi et al., 1997; Zhang et al., 2001; Balestrieri et al., 2008). Of note, this cell line was recently demonstrated to produce poorly infectious viral particles, even when these cells were used for viral transfer after cell-to-cell contacts (Alais et al., 2015). We have developed an easy-to-use assay relying on the co-culture between HTLV-1-infected C91PL T-cells and LTR-luciferase reporter Jurkat T-cells (Pais-Correia et al., 2010). Here, we demonstrate that this assay is useful to screen a series of drugs, in experimental settings that most closely model the *in vivo* viral transmission mode. Our results using stavudine d4T and lamivudine 3TC are consistent with the literature and show that, for some unexplained reasons, these drugs have no effect on HTLV-1 RT. More interestingly, we show here for the first time that Adefovir (PMEA) as well as its prodrug (Adefovir dipivoxil) work much better than AZT. In the case of Tenofovir (PMPA), the drug has no effect, however, the prodrug (Tenofovir disoproxil) has a significant inhibitory effect when used at 10 μM. This encourages us to validate its inhibitory potential *in vivo*, in naturally infected and currently asymptomatic STLV-1 infected non-human primates. The ongoing pre-clinical test combines Tenofovir disoproxil fumarate (Viread) to valproate, in a protocol similar to that used in the Afonso study (Afonso et al., 2010), in which the combination of AZT with valproate led to a strong PVL decrease as long as the treatment did go on. From these results and the results of the present study, it can therefore be expected to get better results with Tenofovir disoproxil fumarate (Viread). This could help HTLV-1 infected patients’ treatment in the future.

## Author Contributions

AP and SA performed the experiments. LR and KA synthesized all phosphonates derivatives except the bis-POC-PMPA fumarate and PMPA Alafenamide which were purchased from Selleckchem. M-IT contributed with technical developments for some experiments and discussion. RM, HD, and KA designed the experiments. RM, CJ, HD, and KA wrote the article.

## Conflict of Interest Statement

The authors declare that the research was conducted in the absence of any commercial or financial relationships that could be construed as a potential conflict of interest.
